# Differences in the organization of the primary motor cortex in people with and without low back pain and associations with motor control and sensory tests

**DOI:** 10.1007/s00221-024-06844-5

**Published:** 2024-05-20

**Authors:** Sabrine P. Klerx, Sjoerd M. Bruijn, Michel W. Coppieters, Henri Kiers, Jos W.R. Twisk, Annelies L. Pool-Goudzwaard

**Affiliations:** 1 Faculty of Behavioural and Movement Sciences, Vrije Universiteit, Amsterdam, The Netherlands; 2 Research Group Lifestyle and Health, HU University of Applied Sciences, Utrecht, The Netherlands; 3 Institute of Brain and Behaviour , Amsterdam, The Netherlands; 4 School of Health Sciences and Social Work, Menzies Health Institute Queensland, Brisbane and Gold Coast, Griffith University, Brisbane and Gold Coast, Australia; 5 Research Centre for Digital Business and Media, HU University of Applied Sciences, Utrecht, The Netherlands; 6 Department of Epidemiology and Data Science, Amsterdam University Medical Centre, Amsterdam, The Netherlands; 7 SOMT University of Physiotherapy, Amersfoort, The Netherlands

**Keywords:** Low back pain, Transcranial magnetic stimulation, Brain mapping, Movement, Motor skills

## Abstract

Differences in organization of the primary motor cortex and altered trunk motor control (sensing, processing and motor output) have been reported in people with low back pain (LBP). Little is known to what extent these differences are related. We investigated differences in 1) organization of the primary motor cortex and 2) motor and sensory tests between people with and without LBP, and 3) investigated associations between the organization of the primary motor cortex and motor and sensory tests. We conducted a case-control study in people with (N=25) and without (N=25) LBP. The organization of the primary motor cortex (Center of Gravity (CoG) and Area of the cortical representation of trunk muscles) was assessed using neuronavigated transcranial magnetic stimulation, based on individual MRIs. Sensory tests (quantitative sensory testing, graphaesthesia, two-point discrimination threshold) and a motor test (spiral-tracking test) were assessed. Participants with LBP had a more lateral and lower location of the CoG and a higher temporal summation of pain. For all participants combined, better vibration test scores were associated with a more anterior, lateral, and lower CoG and a better two-point discrimination threshold was associated with a lower CoG. A small subset of variables showed significance. Although this aligns with the concept of altered organization of the primary motor cortex in LBP, there is no strong evidence of the association between altered organization of the primary motor cortex and motor and sensory test performance in LBP. Focusing on subgroup analyses regarding pain duration can be a topic for future research.

**Supplementary Information:**

The online version contains supplementary material available at 10.1007/s00221-024-06844-5.

## Introduction

Differences in the organization of the primary motor cortex have been reported between people with and without low back pain (LBP) (Chang et al. 2019; Elgueta-Cancino et al. 2017; Jenkins et al. 2021, 2023; Schabrun et al. 2017; Tsao et al. 2008, 2010, 2011). Transcranial magnetic stimulation (TMS), a non-invasive procedure that uses magnetic fields to stimulate nerve cells in the brain, is often used to study the organization of the primary motor cortex. TMS studies revealed changes in cortical map volume (i.e., the cortical volume from which a muscle can be stimulated) (Chang et al. 2019; Tsao et al. 2011), overlap of cortical areas (the cortical area from which stimulations can be elicited)(Tsao et al. 2011) and center of gravity (CoG, the center of the cortical area from which a muscle can be stimulated)(Elgueta-Cancino et al. 2017; Li et al. 2021; Schabrun et al. 2017; Tsao et al. 2011) for various trunk muscles in people with LBP. For instance, compared to people without LBP, the CoG of the longissimus is reported to be both more posterior(Tsao et al. 2011) or more anterior(Schabrun et al. 2017; Elgueata-Cancino et al., 2017) in people with LBP, and for the multifidus more posterior(Li et al. 2021) in people with LBP. The location of the CoG of the transversus abdominis was located more posterior and lateral in patients with LBP (Tsao et al. 2008; Li et al. 2021). Though it is believed that reorganization of the primary motor cortex is a characteristic of LBP, this has not been clearly established. Findings vary and may even be inconsistent between studies (Chang et al. 2019; Elgueta-Cancino et al. 2017; Li et al. 2021; Schabrun et al. 2017; Tsao et al. 2008, 2011).

Altered trunk motor control has been linked to differences in the organization of the primary motor cortex (Massé-Alarie et al. 2016; Tsao et al. 2008, 2010). For example, compared to people without LBP, delayed activation of the transversus abdominis muscle during rapid arm movements was observed in people with LBP (Tsao et al. 2008). This delay was associated with a posterior and lateral shift of the CoG of the transversus abdominis muscle (Tsao et al. 2008). After two weeks of motor training, the transversus abdominis muscle was activated earlier, which was associated with an anterior and medial shift of the CoG of the transversus abdominis muscle (Tsao et al. 2010). Most studies that identified an association between the organization of the primary motor cortex and altered motor control in LBP have focused on latency of muscle activation. However, rapid arm movement and abdominal drawing-in maneuvers are only one aspect of motor control. It is largely unknown how changes to the organization of the primary motor cortex may be associated with other motor control tasks, especially when the motor control tasks are more complex.

Motor control requires accurate multisensory sensing, including proprioceptive and tactile somatosensory inputs, processing, and motor output (Chiba et al. 2016). Multisensory inputs are utilized to estimate the state of the body, subsequently influencing motor behaviour (Chiba et al. 2016). In LBP, sensing (of input), processing and output, may be impaired (Adamczyk et al. 2018; Biely et al. 2014; Elgueta-Cancino et al. 2015; Jung et al. 2020; Luomajoki and Moseley 2009; Tong et al. 2017; Wand et al. 2010) and may be relevant to assess in a clinical setting. Several studies showed that people with LBP performed worse on motor control tests (Biely et al. 2014; Elgueta-Cancino et al. 2015; Jung et al. 2020; Luomajoki and Moseley 2009; Tong et al. 2017) and sensory accuracy tests (Adamczyk et al. 2018; Luomajoki and Moseley 2009; Wand et al. 2010). However, to the best of our knowledge, the association between motor tests and sensory accuracy tests with the organization of the primary motor cortex in people with LBP has only been investigated in two studies (Elgueta-Cancino et al. 2017; Shraim et al. 2023). No associations were found between a motor test, two-point discrimination threshold and map volume, and CoG (Elgueta-Cancino et al. 2017) and between a motor test and cortical excitability and intracortical mechanisms (facilitation and inhibition) at baseline or after motor training (Shraim et al. 2023). Although there is evidence indicating alterations in different components of motor control, associations between this broad aspect of motor control and the organization of the primary motor cortex remain largely unexplored.

Besides the differences in the organization of the primary motor cortex between groups with and without LBP, altered organization of the primary motor cortex of trunk muscles in people with LBP has been associated with the intensity of LBP (Elgueta-Cancino et al. 2017, 2021; Jenkins et al. 2021, 2023; Tsao et al. 2011). However, conflicting findings have been described regarding this association. Several studies found that a smaller longissimus cortical map volume was associated with a higher intensity of LBP (Elgueta-Cancino et al. 2017, 2021; Schabrun et al. 2017), but this was not always observed (Tsao et al. 2011). Similarly, findings regarding the association between the CoG of the longissimus muscle and pain intensity varied among studies (Elgueta-Cancino et al. 2017, 2021; Jenkins et al. 2023; Tsao et al. 2011). One study investigated the association between a pain sensitivity test (pressure pain threshold) and organization of the primary motor cortex. They found an association between the pressure pain threshold and the CoG location of the longissimus, but not with cortical map volume (Elgueta-Cancino et al. 2017). Pain intensity might be an important variable related to the organization of the primary cortex. However, findings are conflicting regarding this association (Elgueta-Cancino et al. 2017, 2021; Schabrun et al. 2017; Tsao et al. 2011; Jenkins et al. 2023).

In summary, conflicting evidence exists regarding the organization of the primary motor cortex and there is limited information about the link between organization of the primary motor cortex and the different components of motor control, including sensing, processing and motor output, and pain modulation in people with LBP. Consequently, there is a need to acquire additional information and gain a more thorough understanding of this association. Therefore, the aims of this study were to explore differences in the organization of the primary motor cortex of trunk muscles, and motor and sensory tests between people with and without LBP, and to explore the association between organization of the primary motor cortex, motor and sensory tests, and pain modulation.

## Methods

We conducted a case-control study with a cross-sectional and longitudinal part in people with and without LBP. The protocol was registered in the Open Science Framework (DOI 10.17605/OSF. IO/5C8ZG) (Klerx et al. 2022). This manuscript reports the cross-sectional part, and the longitudinal part will be published elsewhere. The STROBE checklist for reporting observational studies has been followed. Ethical approval was obtained from METC Brabant (NL70934.028.19). All participants provided written informed consent before commencing the study.

### Participants

**Fig. 1:**
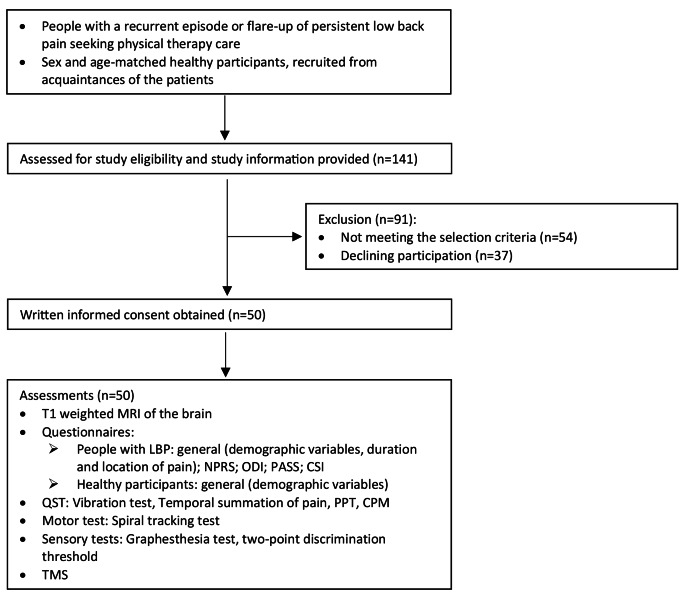
Flowchart of the study. MRI, Magnetic Resonance Imaging; TMS; Transcranial Magnetic Stimulation; LBP, Low Back Pain; NPRS, Numeric Pain Rating Scale; ODI, Oswestry Disability Index; PASS, Pain Anxiety Symptom Scale; CSI, Central Sensitization Inventory; PPT, Pressure Pain Threshold; CPM, Conditioned Pain Modulation 6,25°

People with LBP were recruited from five primary care physical therapy clinics in The Netherlands. Twenty-five participants with LBP were included. All LBP participants had a history of LBP. Eleven experienced chronic LBP with a flare-up duration of a median of 6 days interquartile range (IQR) 7 (n = eight, for three participants this data from the questionnaire was missing); while 14 had a recurrence of LBP with a median of 14 days IQR 22. The median number of pain episodes in the past three years was 4 IQR 5 and the median duration of remission in months was 6 IQR 9 (*n* = 13, for one participant this data from the questionnaire was missing). The flare-up and recurrence were of a duration of at least 24 h at the time of recruiting. All participants were seeking care for their LBP and the experienced LBP had to affect daily activities. With these selection criteria, we aimed to include a group of people with LBP who were representative and who experienced fluctuations in pain and function, with an increase in pain and an impact on daily activities at the time of testing. Because of the longitudinal part of the study, we opted to include participants who had a reasonable chance of recovery over a five-week period. See Table 1 for demographic information of the participants. Twenty-five age and sex-matched participants without LBP were included and were recruited from the acquaintances, friends, or relatives of the patients. Exclusion criteria for both groups included major spinal pathology (e.g. ankylosing spondylitis), a history of lumbar radiculopathy or spinal operation, cardiovascular diseases, or pregnancy and the six-month postpartum period, younger than 18 or older than 65 years of age, or not meeting the safety criteria for Magnetic Resonance Imaging (MRI) or TMS (Rossi et al. 2011). See Fig. 1 for a flowchart of the study.

**Table 1:** Characteristics of the groups

	Participants with LBP (*n* = 25)	Participants without LBP (*n* = 25)
Age	40 (15)	41 (14)
Sex Male: Female	12:13	12:13
BMI	26 (3)	25 (3)
Hemisphere Left: Right	9:16	9:16
NPRS - Current - Max past week - Average past week	4 (2) 7 (2) 4 (1)	
ODI	18 (12)	
PASS	45 (18)	
CSI	26 (10)	

LBP, low back pain, experiencing a flare-up or recurrence of pain (> 24 h) between the lower rib margins and the buttock creases; BMI, body mass index; NPRS, numeric pain rating scale; ODI, oswestry disability index; PASS, pain anxiety symptom scale; CSI, central sensitization inventory; NPRS, ODI, PASS and CSI were only assessed in participants with LBP.

The sample size was calculated based on a cross-sectional comparison between two groups (Schabrun et al. 2017), using CoG as the primary outcome for the organization of the primary motor cortex, with a power of 0.80 and α < 0.05. The calculation produced a required sample size of *N* = 21 per group. A few extra participants per group were included to account for possible exclusions due to incomplete data.

Between 15 October 2020 and 20 July 2021, 25 participants with and 25 participants without LBP were included in the study. Ninety-one additional volunteers registered to participate but did not meet the selection criteria, see Fig. 1. The mean (SD) age for the LBP participants was 40 (15) years and for the participants without LBP 41 (14) years. In both groups, 12 men and 13 women were included (see Table 1).

## Assessments

Participants performed several quantitative sensory tests, and motor and sensory tests. The organization of the primary motor cortex was determined using TMS. The participants with LBP completed the following reliable and valid self-reported questionnaires: a Numeric Pain Rating Scale (0–10) for pain intensity (Williamson and Hoggart 2005), Oswestry Disability Index (Van Hooff et al. 2015), Pain Anxiety Symptom Scale (Lundberg et al. 2011) and Central Sensitization Inventory (Scerbo et al. 2018).

### Quantitative sensory testing

Four tests from the quantitative sensory test battery were performed according to recommended protocols (German Research Network on Neuropathic Pain 2010; Rolke et al. 2006): (1) Vibration sense was assessed over the spinous process of L4 using a Rydel-Seiffer tuning fork (64 Hz, 8/8 scale; US Neurologicals, WA) and was determined based on when the participant indicated that vibration could no longer be felt. The mean of three consecutive trials was used to calculate the vibration threshold (German Research Network on Neuropathic Pain 2010; Rolke et al. 2006; Whitton et al. 2005); (2) Pressure pain threshold was assessed by applying pressure with a hand-held digital algometer (Wagner Instruments Model FDX-25, Greenwich, USA) over the paraspinal musculature at the level L5 on the (most) painful side. The pressure was increased at a rate of ~ 5 N/s. The participant was asked to indicate when the sensation of pressure changed to a sensation of painful pressure. The threshold was based on the mean of three repetitions protocols (German Research Network on Neuropathic Pain 2010; Rolke et al. 2006); (3) Conditioned pain modulation was assessed with the cold pressor test. Pressure pain thresholds (as described above) were assessed before and while the right hand was submerged in cold water (10 degrees). Three PPT measures were performed at the same location as the baseline PPT measures, with 30s rest periods between PPTs. Numeric Pain Rating Scale scores for the submerged hand pain were recorded following each PPT. The relative and absolute conditioned pain modulation effect were calculated as recommended (Reezigt et al. 2021; Yarnitsky et al. 2015) and (4) Temporal summation of pain was measured by first applying a single stimulus with a 256 mN pinprick (MRC-systems GmbH, Heidelberg), followed by a train of 10 stimuli paraspinal to L5 on the (most) painful side. This was performed five times, with 1-min rest in between repetitions (German Research Network on Neuropathic Pain 2010; Rolke et al. 2006). The mean pain intensity following a single stimulus was subtracted from the mean pain intensity following the train of 10 stimuli to calculate the temporal summation of pain effect.

### Sensory tests

Graphaesthesia (Klerx et al. 2022; Wand et al. 2010) was tested with the participant lying prone. The participant had to recognize 20 numbers drawn on the lower back with the back of the monofilament holder (Wand et al. 2010). The outcome was the error rate, which was calculated by dividing the number of incorrect answers by 20. Sensory discrimination was also assessed by determining the two-point discrimination threshold paraspinal to L1, L3 and L5 (six locations), using a 2-point discriminator (Carolina Biological Supply Company, Burlington, NC, USA), with the participant lying prone (Ehrenbrusthoff et al. 2016; Klerx et al. 2022). The test started with the calipers at 20 mm distance horizontally, which was increased in five mm steps as long as the participant identified only one stimulus. The outcome was determined by computing the mean of the averages of the distance in mm when two points were identified in each of the six locations.

### Motor test

**Fig. 2:**
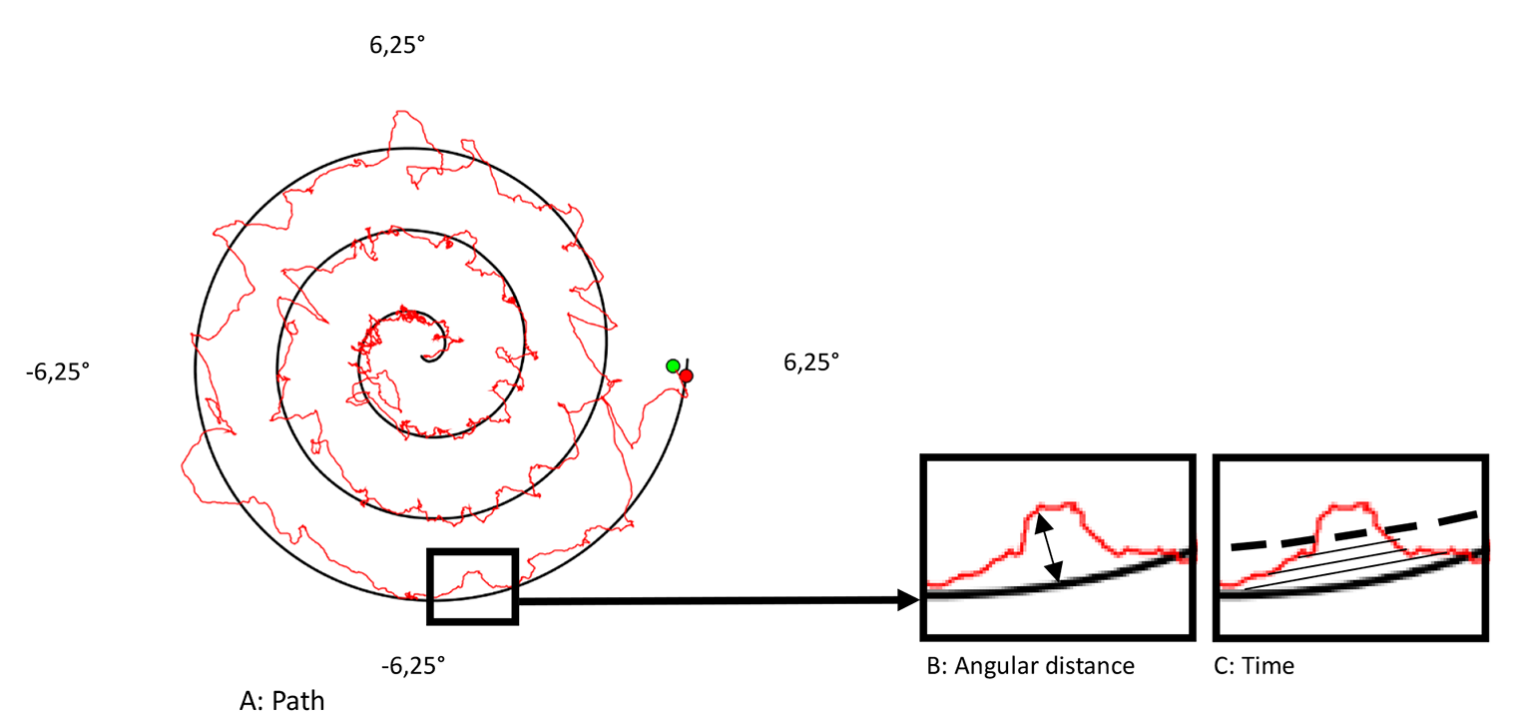
Parameters measured in the spiral tracking test: Reprinted and modified with permission from Klerx et al. 2023(Klerx et al. 2023). The tracking error was calculated based on the absolute difference between the target angle and the actual inclination angle of the trunk in the frontal (x) and sagittal (y) axis of motion in degrees. The orientation of the sensor in degrees of the x and y axis was converted on a 2 dimensional screen, calculated as $\surd ( {\left(targe{t}_{x}-actua{l}_{x}\right)}^{2 }+{\left(targe{t}_{y}-actua{l}_{Y}\right)}^{2})$. The spiral on the screen was set at 13,5 × 13,5 degrees (the x axis − 6,25 degrees to 6,25 degrees and the y axis − 6,25 to 6,25 degrees). Panel A: Path, the red squiggly line represents the sum of alle differences between the target position and the actual position. Panel B: Angular distance, the black arrow represents the error as the distance in degrees from the red target point (the spiral). Panel C: Time, the lines till the dashed lines represent the area of the time spent in 0,9° distance from the red target point (the spiral)

Movement precision was assessed with a spiral tracking test (Klerx et al. 2023). In this task, the participant was asked to move the trunk, from an upright sitting position, to steer a green point (indicating the orientation of a movement sensor (EN-14,001–9DoF Razor IMU M0 Accelerometer and Motion Sensor Breakout, SparkFun Electronics, USA) and a microcontroller (SAMD21G18A), on a computer monitor as closely as possible to a red target point. The red target point moved anticlockwise along the lines of a spiral. The sensor was attached to the skin at the level of T12. The task took about two minutes to complete and was performed twice. The first trial was considered a practice trial. The tracking error was calculated based on the absolute difference between the target angle and the actual inclination angle of the trunk in the sagittal (x) and transversal (y) axis in degrees (Willigenburg et al. 2013). As outcome for motor control we calculated (1) the mean of the closest 90% tracking errors; (2) the mean percentage of the total time spent at an angular distance closer than 0,9° from the red target point and (3) the path, the sum of all differences between the target position and the actual position (Klerx et al. 2023), see Fig. 2. Data from the sensors were analyzed using custom-written Matlab scripts (R2014B, The MathWorks, Natick, MA). A detailed description of the outcome measures has been published elsewhere (Klerx et al. 2023).

### Organization of the primary motor cortex

For precise navigation of TMS (Ruohonen and Karhu 2010), each participant underwent a T1-weighted MRI scan of the brain (SIEMENS MAGNETOM Vida-XQ-32 Numaris/X VA20A-04 ML). Whole-brain grey matter was segmented using SPM12 (*SPM*, n.d.) for use in neural navigation (Neural Navigator 3.4, BrainScience Tools, The Netherlands). In line with the existing literature (Elgueta-Cancino et al. 2017; Jenkins et al. 2021, 2023; Schabrun et al. 2017), we opted for surface electromyography (EMG) to record muscle activity of the longissimus muscle at the level of L3 and L5, and of the obliquus externus and internus, since indwelling intramuscular EMG electrodes may be uncomfortable with trunk movements which may affect the outcome. Disposable, bipolar pre-gelled rectangular ECG electrodes (AG/AgCl Ambu Blue Sensor N, Medicotest, Ølstykke, Denmark) were placed according to SENIAM recommendations (Stegeman and Hermens 2007). EMG was captured using a 16-channel Porti EMG device (Twente Medical Systems International B.V., Enschede, The Netherlands). To lower the threshold at which Motor Evoked Potentials (MEP) could be evoked, pre-activation at 20% MVC of the longissimus was asked during the measurements (Cavaleri et al. 2020; Elgueta-Cancino et al. 2017; Jenkins et al. 2021; Schabrun et al. 2014; Tsao et al. 2011). Pre-activation was obtained by having the participant sit upright and when required resist a weight connected to a custom Velcro Harness via a pulley system, which pulled the participant horizontally. Visual feedback about the generated force and the target force was provided on a computer screen. A Magstim 200^2^ stimulator (Magstim Company Ltd., Whitland, Dyfed, UK), combined with a figure-of-eight coil with 70 mm windings was used to deliver a single-pulse TMS to the hemisphere contralateral to the side of the highest pain intensity. The coil was orientated 45 degrees to the sagittal plane (Jin et al. 2022). The stimulation intensity was set at 100%, as 120% of the motor threshold of the longissimus muscle generally exceeded the maximum output of the stimulator (Elgueta-Cancino et al. 2017; Schabrun et al. 2017). During the TMS protocol, the EMG of the longissimus at level L3 of the most painful site was monitored for MEPs. We used a protocol with 100 stimulations at pseudorandom positions (Cavaleri et al. 2020; Van De Ruit et al. 2015), covering the area of the motor cortex. When MEPs were still elicited at the borders of motor cortex stimulations were given in the surroundings, to make sure the area from which MEPS could be elicited was completely covered. The measurements were conducted according to the TMS checklist for methodological quality (Fuentes et al. 2012).

The data of the neural navigation, combined with the EMG, were analyzed in Matlab (R2019b) in accordance with Jin et al. 2022 (Jin et al. 2022). The EMG data were high pass filtered at 30 Hz, and MEPs were defined as 500ms epochs following a stimulation in which the peak-to-peak amplitude was > 50µV. Values less than 25% of the peak response were removed. To correct for differences in the shape and the size of an individual’s brain, individual stimulation locations were warped to a MNI template (Kraus and Gharabaghi 2015). Then, we calculated for each muscle of the (most) painful side (longissimus level L3 and L5, obliquus externus and internus 1) the CoG, using the formula: CoG = Σ(Vi x Xi) /Σvi ; Σ(Vi x Yi)/Σvi, ; Σ(Vi x Zi)/Σvi, where: Vi = MEP amplitude at site i, which has the coordinates Xi, Yi, Zi (Tsao et al. 2011) and 2) the cortical area from which stimulations could be elicited, by means of a custom-written algorithm (see Jin et al. 2022 (Jin et al. 2022) and https://github.com/marlow17/surfaceanalysis). We did not analyze cortical map volume, since this variable is based on amplitudes, which are prone to noise. Moreover, the map volume requires that stimulations are given at pre-defined locations, which was not the case with our random stimulation protocol.

### Statistical analysis

Multivariate mixed model analyses with a 2-level structure were used to analyze differences between the groups in (1) organization of the primary motor cortex (CoG and area) of trunk muscles (i.e., longissimus at level L3 and L5, obliquus externus and internus) and (2) the motor and sensory tests. Multivariate mixed model analysis was used because the outcomes on the different tests are correlated within the participant. Thus, outcomes of the different tests were analyzed together in one model. To adjust for this correlation, a random intercept on subject level was added to the model. Furthermore, the model contained a Group variable, a Test variable (i.e., a categorical variable which indicates which outcome belongs to a particular test) and the interaction between the Group variable and the Test variable. In this way, the coefficients with standard error, 95% Confidence Interval and *P* value were analyzed per variable within this model, indicating the difference between the group with and without LBP per test variable. For the organization of the primary motor cortex, two models were used, one model for the CoG and one model for the cortical area. Within these two models, the test variable included the trunk muscles (i.e., longissimus at level L3 and L5, obliquus externus and internus). For the sensory accuracy tests, the test variable included vibration, graphaesthesia and two-point discrimination threshold. The test variable for quantitative sensory testing included temporal summation of pain, pressure pain threshold, and relative and absolute conditioned pain modulation. Finally, for the spiral tracking test, the test variable included the parameters angular distance closer than 0,9°, time spent at an angular distance closer than 0,9° and path. To analyze the association for all participants together, between the organization of the primary motor cortex of trunk muscles and the performance on motor control and sensory tests, the Test variable of the organization of the primary motor cortex was used as multivariate outcome. This was analyzed on association with each motor and sensory test variable. When a significant association was demonstrated, this association was subsequently analyzed within each group. Multivariate analyses managed dependencies of the observations between the outcome within the participant and accounted for multiple testing to a certain degree because all estimates were derived from the same model. Considering the exploratory nature of this research, in addition to analyzing statistical significance, we were also interested in uncovering patterns. Therefore, we opted not to implement additional corrections for multiple testing. All multivariate multilevel analyses were performed with STATA (version 17).

## Results

### Missing data

Due to the fact that not for all participants MEPs could be elicited for all muscles, there were missing data in the outcomes for cortical organization. For the entire dataset, 13 muscle outcomes were missing from nine participants (four with LBP and five without LBP; longissimus L3: seven; longissimus L5: three; obliquus externus: zero; obliquus internus: three). For each muscle group, there were data outcomes for 50 participants, resulting in a comprehensive dataset for a total of 200 primary outcomes across all four muscles. There was 6,5% of missing data for the entire dataset. There were no missing data in the outcomes of the clinical tests.

### Multivariate analyses

Through visual inspection, it was observed that the residuals of all models exhibited an approximately normal distribution and no signs of heteroscedasticity were evident.

### Organization of the primary motor cortex

**Fig. 3:**
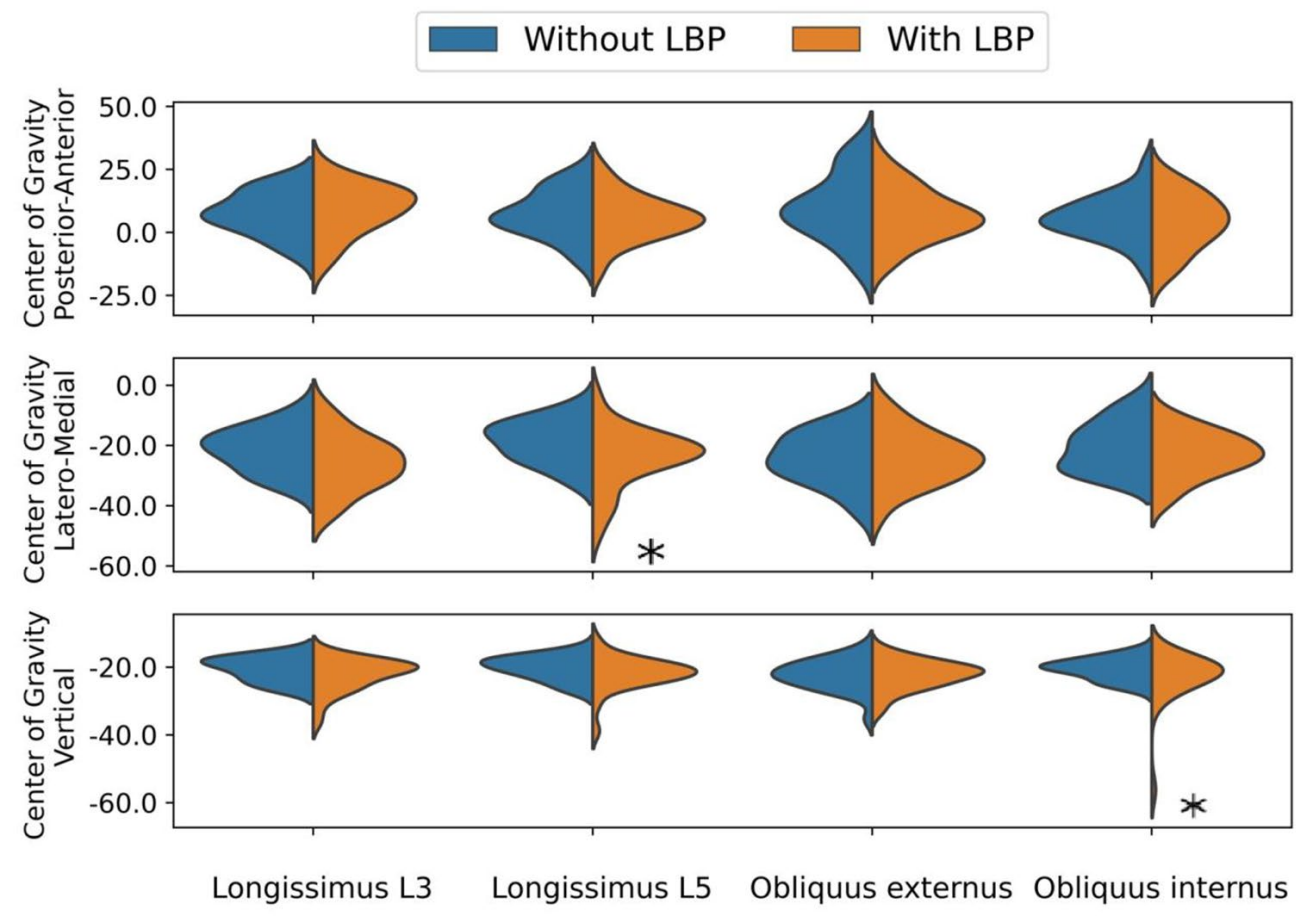
The distribution of the Center of Gravity for people with and without Low Back Pain. Within these multivariate mixed model analyses, significant differences are highlighted with an asterisk (*)

The location of the CoG of the longissimus at level L5 was significantly more lateral in the LBP group compared to the group without LBP, and for the CoG for the obliquus internus lower in the vertical direction, see Fig. 3; Table 2. No other significant differences were found for the CoG (see Table 2). We found no significant differences in area between groups (see Table 2).

**Table 2:** Multivariate analyses of the differences between groups in TMS assessments

Dependent variable	Muscle	β_group_ (SE)	95%CI	*P* value
Area	Longissimus L3	-363.586 (630.255)	-1598.863-871.691	0.564
	Longissimus L5	234.646 (614.875)	-970.486-1439.778	0.703
	Obliquus externus	1050.963 (604.249)	-133.343-2235.269	0.082
	Obliquus internus	3.591 (614.875)	-1201.541-1208.723	0.995
Center of Gravity	Longissimus L3	-0.622 (2.898)	-6.302-5.058	0.830
Anterior-posterior	Longissimus L5	-1.989 (2.843)	-7.561-3.583	0.484
	Obliquus externus	-2.887 (2.807)	-8.387-2.614	0.304
	Obliquus internus	-2.064 (2.842)	-7.634-3.505	0.468
Center of Gravity	Longissimus L3	-4.120 (2.430)	-8.883-0.644	0.090
Medio-lateral	**Longissimus L5**	**-5.916 (2.376)**	**-10.573- -1.259**	**0.013**
	Obliquus externus	0.202 (2.340)	-4.385-4.789	0.931
	Obliquus internus	-2.807 (2.375)	-7.462-1.847	0.237
Center of Gravity	Longissimus L3	-1.298 (1.420)	-4.082-1.486	0.361
Vertical	Longissimus L5	-2.231 (1.379)	-4.934-0.472	0.106
	Obliquus externus	-0.002 (1.352)	-2.652-2.649	0.999
	**Obliquus internus**	**-2.709 (1.379)**	**-5.411- -0.007**	**0.049**

The coefficients can be interpreted as the difference in one unit in the variable for the people with low back pain compared to the people without low back pain. Statistically significant values are highlighted in bold

### Clinical tests

Participants with LBP exhibited a significant higher temporal summation of pain compared to participants without LBP (Table 3). We found no significant differences between participants with and without LBP for other quantitative sensory tests, sensory accuracy tests or the motor test (Table 3).

**Table 3:** Multivariate analyses of the differences between groups in clinical assessments

Dependent variable	Test	β_group_ (SE)	95%CI	*P* value
QST, pain	**Temporal summation**	**0.992 (0.341)**	**0.323–1.661**	**0.004**
	PPT	-6.096 (6.157)	-18.163-5.971	0.322
	CPM relative	5.351 (6.157)	-6.717-17.418	0.385
	CPM absolute	-2.323 (6.157)	-14.390-9.745	0.786
Sensory accuracy	Two Point Discrimination	1.533 (4.511)	-7.307-10.374	0.734
	Graphaesthesia	1.400 (4.511)	-7.441-10.241	0.756
	Vibration	0.027 (0.226)	-0.415-0.469	0.906
Motor test;	Path	-8.588 (8.380)	-25.014-7.837	0.305
spiral tracking test	Angular distance_Near	0.029 (0.084)	-0.136-0.193	0.733
	Time_Near	-1.203 (4.190)	-9.416-7.010	0.774

The coefficients can be interpreted as the difference in one unit in the variable for the people with low back pain compared to the people without low back pain. QST, Quantitative Sensory Testing; PPT, pressure pain threshold; CPM, conditioned pain modulation; Path, the total distance travelled in degrees over one trial, and calculated over each quadrant; Angular distance_Near: the mean of the closest 10% to the closest 90% tracking errors; Time_Near: the mean percentage of time spent at an angular distance closer than 0,1° to 0,9° from the red target point. Statistically significant values are highlighted in bold

### Association between the organization of the primary motor cortex and clinical tests

**Fig. 4:**
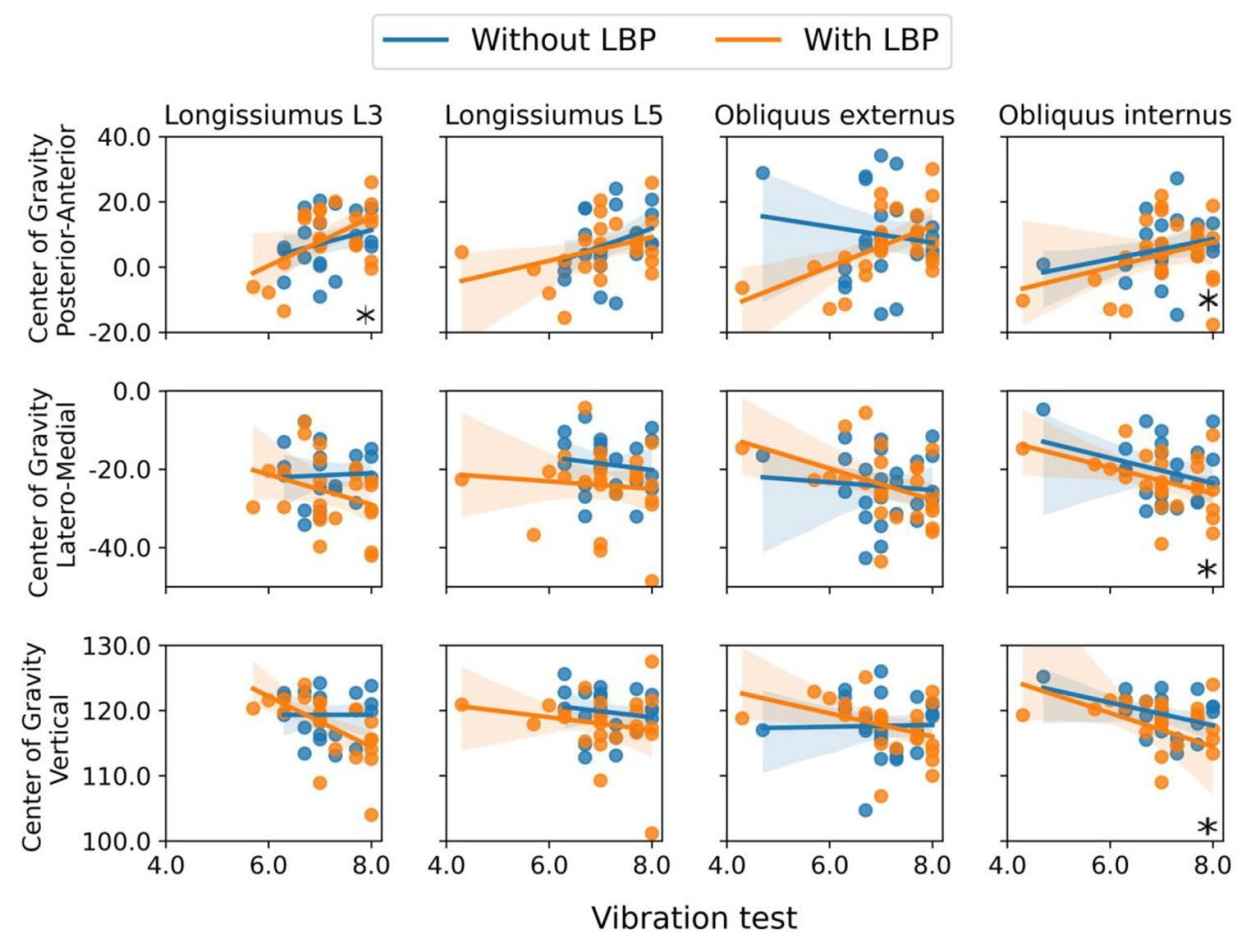
Results of the significant associations between Center of Gravity and the vibration test. Within these multivariate mixed model analyses, significant associations are highlighted with an asterisk (*)

**Fig. 5:**
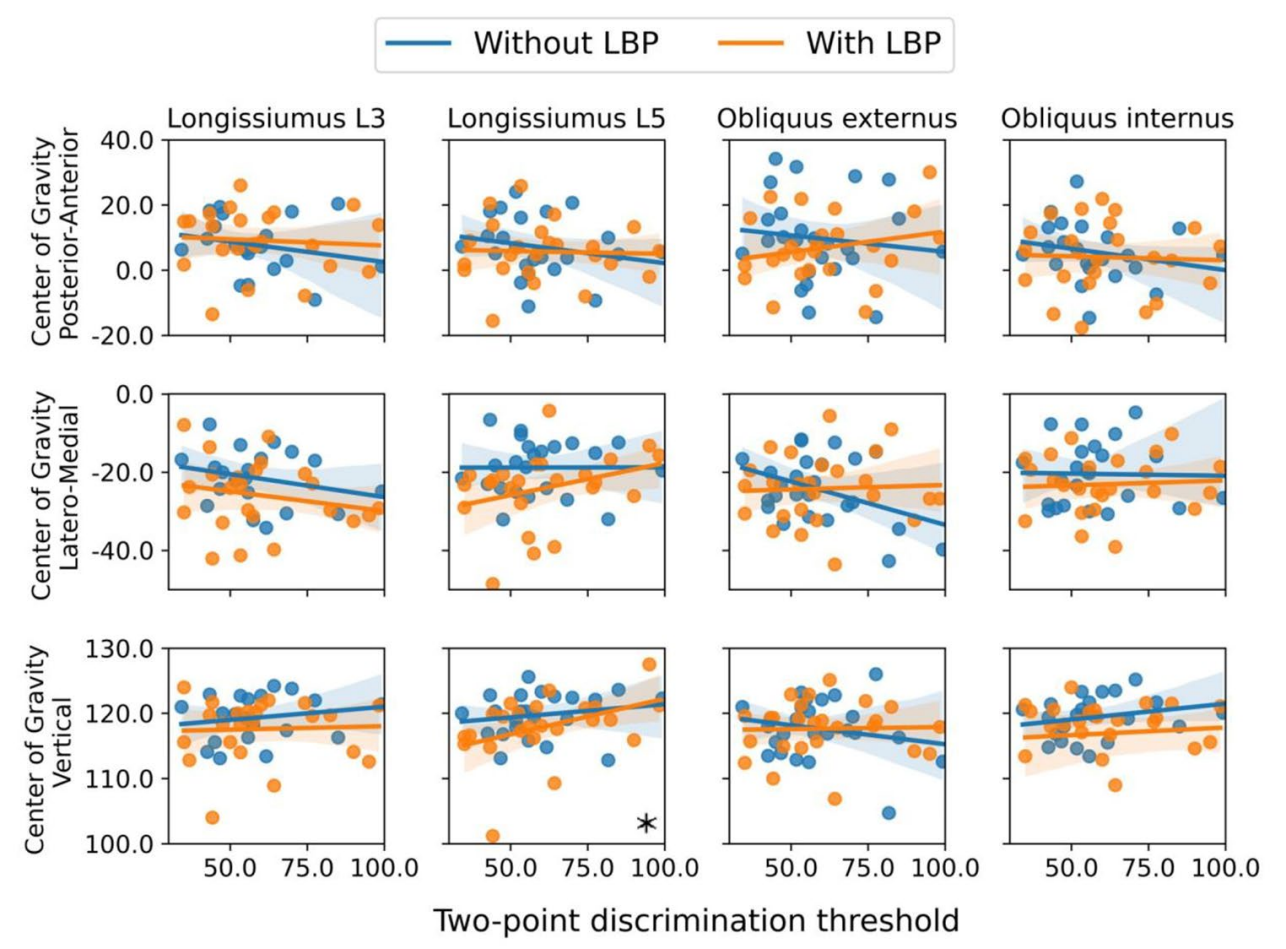
Results of the significant associations between Center of Gravity and the two-point discrimination threshold. Within these multivariate mixed model analyses, significant associations are highlighted with an asterisk (*)

For all participants combined (i.e., people with and without LBP together), a better score on the vibration test (i.e., being able to feel smaller vibrations) was significantly associated with a location of the CoG that was (1) more anterior for the longissimus muscle at L3 and for the obliquus internus; (2) more lateral for the obliquus internus and (3) lower for the obliquus internus, see Fig. 4; Table 4. In addition, a better score on the two-point discrimination threshold (i.e., being able to identify two points as being separate over a smaller distance) was significantly associated with a lower CoG of the longissimus at L5, see Fig. 5; Table 4.

**Table 4:** Multivariate analyses of the significant associations between CoG and clinical assessments

Dependent variable	Muscle	Independent variable	Independent variable	β_association_ (SE)	95%CI	*P* value
Center of Gravity		Sensory accuracy	Group			
Anterior-posterior	Longissimus L3	Vibration	**All** Without LBP **LBP**	**4.917 (2.008)** 2.171 (3.181) **6.881 (2.451)**	**0.981–8.854** -4.065-8.406 **2.078–11.684**	**0.014** 0.495 **0.005**
	Obliquus internus	Vibration	**All** Without LBP LBP	**3.448 (1,736)** 3.086 (2.648) 3.768 (2.173)	**0.046–6.851** -2.105-8.277 -0.492-8.027	**0.047** 0.244 0.083
Medio-lateral	Obliquus internus	Vibration	**All** Without LBP **LBP**	**-3.470 (1,485)** -1.502 (2.940) **-4.041 (1.863)**	**-6.381–0.558** -6.995-1.917 **-7.693–0.389**	**0.019** 0.264 **0.030**
Vertical	Longissimus L5	Two Point Discrimination	**All** Without LBP **LBP**	**0.425 (0.210)** 0.226 (0.321) **0.587 (0.264)**	**0.015–0.835** -0.403-0.855 **0.069–1.106**	**0.042** 0.481 **0.026**
	Obliquus internus	Vibration	**All** Without LBP **LBP**	**-2.395 (0.850)** -1.362 (1.301) **1.700 (1.680)**	**-4.060–0.730** -3.911-1.188 **-1.591-4.992**	**0.005** 0.295 **0.004**

Two Point Discrimination is presented in coefficient x 5, as the test increases in steps of 5

Further analysis demonstrated that the above described significant associations were also significant within the LBP group, but not within the group of participants without LBP. However, this was not the case for the association between the vibration test and the more anterior location of the CoG of the obliquus internus. No other significant associations between the organization of the primary motor cortex and motor- and sensory tests were found (see Appendices 1–4).

## Discussion

We studied differences in the organization of the primary motor cortex of trunk muscles, and motor and sensory tests between people with and without LBP, and assessed the association between the organization of the primary motor cortex, motor and sensory tests, and pain modulation. Out of all the analyses we conducted, only a small proportion revealed significant results. We found that people with LBP had a more lateral location of the CoG of the longissimus at L5 and lower CoG of the obliquus internus, as well as higher scores on temporal summation of pain. When scoring better on the vibration test, the CoG was located more anterior (longissimus L3 and obliquus internus), lateral and lower (obliquus internus) and when scoring better on the two-point discrimination threshold, the CoG was located lower (longissimus L5).

We found that people with LBP had a significantly more lateral CoG of the longissimus muscle at L5. This more lateral location is similar to two previous studies, which reported a more lateral location for the transversus abdominis muscle (Tsao et al. 2008) and the multifidus muscle (Li et al. 2021). We found no significant difference in anterior-posterior location of the CoG. Previous studies described both a more posterior (Tsao et al. 2011) and anterior (Elgueta-Cancino et al. 2017; Schabrun et al. 2017) location for the longissimus muscle. We found no differences between groups for the cortical area. This is comparable with a previous study where the area (defined as the sites on the scalp grid from which an MEP was obtained) was analyzed for the transversus abdominis and multifidus muscle (Li et al. 2021). While the majority of cortical outcomes did not reveal statistical significance, participants with LBP tended to have a larger cortical area and a more posterior, lateral and lower location of the CoG. Noteworthy, an analysis conducted in subgroups regarding pain mechanisms (nociceptive/nociplastic/ a combination of nociceptive and nociplastic) revealed changes between groups in area, overlap and a correlation between overlap and severity of LBP for the longissimus and multifidus (Elgueta-Cancino et al. 2021). Overall, our findings partially align with previous literature, supporting the theory that the organization of the primary motor cortex is altered in people with LBP. Subgroup analysis in pain mechanisms regarding cortical organization might be of interest for future investigation.

Among the various clinical tests we administered, we only found a statistically significant difference for temporal summation of pain. This significantly higher score for people with LBP compared to people without LBP is in line with a previous meta-analysis (Den Bandt et al. 2019). For pressure pain threshold and conditioned pain modulation, for which we found no significant difference, the literature reported mixed outcomes (Den Bandt et al. 2019). For the graphaesthesia test, a previous study showed a significant difference between people with and without LBP (Wand et al. 2010). In our study, however, we observed substantially lower error rates. This could be because we opted to employ 20 numbers instead of 60 letters, after we found in pilot studies that the reliability when using letters was limited. Compared to the differences in two-point discrimination threshold reported in the literature (i.e., 6-(Elgueta-Cancino et al. 2017)-16 mm(Ehrenbrusthoff et al. 2018), we found that people with LBP had comparable thresholds, but had only a very small higher threshold (1.5 mm) than people without LBP. This can be explained by the fact that in our study people without LBP already had a relatively high threshold. We have no clear insights why the people without LBP performed worse in our study compared to previous studies in the literature, apart from the fact that people without LBP in our sample were older (Wand et al. 2010). However, despite almost none of the clinical outcomes being significant, people with LBP in general scored worse on clinical tests (but not significantly) than people without LBP (although not for absolute conditioned pain modulation and vibration test).

We found that when scoring better on the vibration test, the CoG of the longissimus L3 was located more anterior and the CoG of the obliquus internus muscle was located more anterior, lateral, and lower. In addition, when scoring better on the two-point discrimination threshold, the CoG of the longissimus L5 was also located lower. Further analysis showed that these associations were present within the LBP group, but not in the group without LBP. Interestingly, these results would mean that for people with LBP, a better vibration or two-point discrimination threshold would coincide with a more ‘abnormal’ (i.e., more deviating from the people without LBP) CoG location in medio-lateral and lower direction. The association we identified between the organization of the primary motor cortex and the vibration test, might potentially be explained by the role of proprioceptive feedback for determining neural control strategies to control the spine, ensuring the facilitation of muscles (Boendermaker et al. 2014). The primary cortex serves as a mediator between modified proprioception and volitional movements, particularly in collaboration with the primary somatosensory cortex (Gandolla et al. 2014). However, the vibration test is executed to mostly exclude proprioceptive information from muscles (by placing the tuning fork over bony prominences). The exact implications of the association we found are currently unclear and require further study and replication.

In previous studies, associations were found between the organization of the primary motor cortex and latency of muscle activation (Tsao et al. 2008, 2010). In our study, we focused on clinical tests. We found only a small subset of significant associations between the organization of the primary motor cortex and clinical tests. One previous study analyzed associations between the organization of the primary motor cortex and clinical tests and found an association between the pressure pain threshold and CoG. Nevertheless, they found no association between a motor test, the two-point discrimination threshold with cortical map volume and CoG (Elgueta-Cancino et al. 2017). Another study also found no associations between a motor test and cortical excitability and intracortical mechanisms (facilitation and inhibition) at baseline or after motor control training (Shraim et al. 2023). The limited number of significant associations between the organization of the primary motor cortex with clinical tests aligns with our overall findings of the absence of differences between people with and without LBP in the clinical tests.

The sample we used in this study was based on a calculation that was derived from a previous study, focusing primarily on the main outcome, the CoG. However, it is important to emphasize that we assessed and analyzed several additional outcomes, such as clinical tests, in this study. Consequently, the sample size we used for the analysis of all outcomes was relatively small. However, we conducted our statistical analysis using multivariate multilevel analysis. This approach allowed us to account for multiple testing to a certain degree (because all estimates were derived from the same model) and because of that, we did not perform an additional adjustment for multiple testing in the present paper. Besides that, with the multivariate multilevel analysis, we managed dependencies of the observations between the outcome within the participant, enabling us to draw exploratory meaningful conclusions from our interrelated dataset.

We adhered to establish research protocols for the set-up and used TMS stimulation at 100% of stimulator output while employing pre-activation of the longissimus muscle (Elgueta-Cancino et al. 2017; Schabrun et al. 2017; Tsao et al. 2011). This decision was made because, in general, setting the stimulation intensity at 120% of the motor threshold for the longissimus muscle surpassed the stimulator’s maximum capacity. However, it is of importance to highlight that due to this standardized intensity, some participants may have experienced a stimulation intensity which was too high, while for others, the stimulation intensity may have been too low to induce MEPs; this may have potentially affected the accuracy of CoG and area estimates. We tried to improve the accuracy of our assessment of the organization of the primary motor cortex in three ways: (1) We used individualized whole-brain anatomical MRI navigation. This enabled us to analyze more individualized, by accounting for variations in participants’ brain shapes and sizes. In this way, the positions of the MEPs could be calculated and for the longitudinal part of our study, using individual MRIs allowed us to analyze the same absolute positions over time; (2) We used a custom 3D analysis method. We employed this method to calculate the representations of muscles within the primary motor cortex, determining the cortical area responsible for muscle excitability. Importantly, it excluded stimulations without motor evoked potentials and avoided assumptions about the underlying geometry; (3) We used a pseudo-random stimulation protocol, which placed stimulations closer together in contrast to grid-based stimulation protocols. This design increased spatial resolution in our outcome measures, enhancing the precision of our investigation into the organization of the primary motor cortex (De Ruit et al., 2014). Brain mapping with TMS, using a pseudo-random stimulation protocol has been found to be reliable and valid (De Ruit et al., 2014). Although our protocol was based on previous research (Elgueta-Cancino et al. 2017; Schabrun et al. 2017; Tsao et al. 2011), and adheres to the TMS checklist (Fuentes et al. 2012), to our knowledge the clinimetric properties regarding brain mapping in trunk muscles are unknown.

In our study, we included participants with both a recurrence of LBP or chronic LBP with a flare-up of pain. The duration of recurrence and flare-up was similar. The participants who experienced a recurrence of pain had a pain-free episode preceding the current episode, whereas participants with chronic pain had continuous pain. To our knowledge, it is unknown how long it takes for LBP to induce maladaptive neuroplasticity. However, the relatively short duration of the flare-up or recurrence may have attenuated the results observed in the organization of the primary motor cortex and may have influenced the number of significant differences for the clinical tests. It is not clear to what extent people with recurrent LBP are fundamentally different from people with continuous LBP. Research in muscle morphology revealed differences between people with continuous LBP and recurrent LBP in remission (Goubert et al. 2017). However, no differences were also reported regarding muscle atrophy in back muscles between participants with continuous chronic pain, non-continuous chronic pain and recurrent pain in remission (Goubert et al. 2017). In our study, we tested the participants with recurrent LBP while experiencing an episode of LBP, not at time of remission as in the study of (Goubert et al. 2017). While it is unknown whether there are differences in the organization of the primary motor cortex between people with recurrent and chronic LBP, focusing on these subgroup analyses can be a topic for future research.

## Conclusion

Among the various variables we analyzed, only a small subset displayed significant results: differences in CoG of specific muscles, differences in temporal summation of pain and an association between vibration, two-point discrimination and CoG. Although this study partially supports the theory that the organization of the primary motor cortex is altered in people with LBP, there is no strong evidence that altered organization of the primary motor cortex is associated with motor and sensory test performance in people with LBP.

### Electronic supplementary material

Below is the link to the electronic supplementary material.

Supplementary Material 1

## Data Availability

Data will be made available on reasonable request.
