# Supplementary material for: Differences in the organization of the primary motor cortex in people with and without low back pain and associations with motor control and sensory tests
Source: Exp Brain Res. 2024 May 20;242(7):1609–22. doi: 10.1007/s00221-024-06844-5 (PMC11208231; doi:10.1007/s00221-024-06844-5)
Supplement: Supplementary file 1 — Supplementary Material 1 [file 221_2024_6844_MOESM1_ESM.docx]

**Appendices**

Appendix 1 Multivariate analyses of the association between area and clinical assessments

| Dependent variable | Muscle | Independent variable | β^­^_association_ (SE) | 95%CI | P value |
| --- | --- | --- | --- | --- | --- |
|  |  | QST pain variables |  |  |  |
| Area | Longissimus L3 | Temporal summation  PPT  CPM relative  CPM absolute | 247.252 (275.532)  10.011 (14.698)  -5.152 (10.646)  -3.479 (12.768) | -292.781-787.284  -18.798-38.819  -26.018-15.714  -28.504-21.545 | 0.370  0.496  0.628  0.785 |
|  | Longissimus L5 | Temporal summation  PPT  CPM relative  CPM absolute | 320.793 (272.900)  7.390 (13.785)  7.092 (10.575)  -14.529 (12.677) | -214.081-855.666  -19.628-34.407  -13.635-27.818  -39.376-10.318 | 0.240  0.592  0.502  0.252 |
|  | Obliquus externus | Temporal summation  PPT  CPM relative  CPM absolute | 201.051 (262.661)  16.252 (13.728)  4.655 (10.456)  -2.105 (12.597) | -313.755-715.858  -10.653-43.158  -15.838-25.148  -26.795-22.585 | 0.444  0.236  0.656  0.867 |
|  | Obliquus internus | Temporal summation  PPT  CPM relative  CPM absolute | 320.654 (267.794)  26.540 (13.800)  1.122 (10.604)  -15.092 (12.719) | -204.213-845.521  -0.507-53.586  -19.661-21.905  -40.022-9.838 | 0.231  0.054  0.916  0.235 |
|  |  | Sensory accuracy |  |  |  |
| Area | Longissimus L3 | Two Point Discrimination  Graphaesthesia  Vibration | -147.404 (94.016)  -108.175 (99.321)  793.055 (459.114) | -331.672-36.864  -302.847-86.497  -106.792-1692.901 | 0.117  0.276  0.084 |
|  | Longissimus L5 | Two Point Discrimination  Graphaesthesia  Vibration | -99.035 (92.220)  36.1445 (98.744)  730.642 (404.039) | -279.783-81.713  -157.391-229.680  -61.259-1522.543 | 0.283  0.714  0.071 |
|  | Obliquus externus | Two Point Discrimination  Graphaesthesia  Vibration | -139.204 (91.253)  49.230 (98.140)  404.035 (376.839) | -318.057-39.649  -134.121-241.581  -334.557-1142.627 | 0.127  0.616  0.284 |
|  | Obliquus internus | Two Point Discrimination  Graphaesthesia  Vibration | 11.151 (93.320)  -75.560 (99.375)  528.693 (380.219) | -171.753-194.055  -270.332-119.211  -216.522-1273.908 | 0.905  0.447  0.164 |
|  |  | Motor control; spiral tracking test | |  |  |
| Area | Longissimus L3 | Path  Angular distance_Near  Time_Near | -7.915 (11.356)  1318.782 (1344.357)  -17.156 (25.123) | -30.171-14.342  -1316.109-3953.673  -66.397-32.085 | 0.486  0.327  0.495 |
|  | Longissimus L5 | Path  Angular distance_Near  Time_Near | 3.287 (9.381)  893.928 (1176.866)  -12.343 (21.355) | -15.100-21.673  -1412.686-3200.543  -54.199-29.512 | 0.726  0.448  0.563 |
|  | Obliquus externus | Path  Angular distance_Near  Time_Near | -3.820 (8.890)  565.814 (1172.959)  -7.899 (21.248) | -21.244-13.605  -1733.144-2864.773  -49.544-33.746 | 0.667  0.630  0.710 |
|  | Obliquus internus | Path  Angular distance_Near  Time_Near | -10.943 (8.917)  -74.223 (1177.451)  4.713 (21.325) | -28.420-6.535  -2381.984-2233.536  -37.083-46.510 | 0.220  0.950  0.825 |

QST, Quantitative Sensory Testing; PPT, pressure pain threshold; CPM, conditioned pain modulation; Path, the total distance travelled in degrees over one trial, and calculated over each quadrant; Angular distance_Near: the mean of the closest 10% to the closest 90% tracking errors; Time_Near: the mean percentage of time spent at an angular distance closer than 0,1° to 0,9° from the red target point. Two Point Discrimination and Graphaesthesia are presented in coefficient x 5, as the tests increase in steps of 5.

Appendix 2 Multivariate analyses of the association between CoG anterior-posterior coordinate and clinical assessments

| Dependent variable | Muscle | Independent variable | β^­^_association_ (SE) | 95%CI | P value |
| --- | --- | --- | --- | --- | --- |
|  |  | QST pain variables |  |  |  |
| CoG  Anterior-posterior | Longissimus L3 | Temporal summation  PPT  CPM relative  CPM absolute | -0.053 (1.261)  0.099 (0.067)  0.041 (0.049)  0.050 (0.059) | -2.997-1.947  -0.033-0.231  -0.055-0.137  -0.065-0.165 | 0.677  0.141  0.401  0.394 |
|  | Longissimus L5 | Temporal summation  PPT  CPM relative  CPM absolute | 0.687 (1.252)  0.064 (0.064)  0.024 (0.049)  0.023 (0.059) | -1.767-3.142  -0.061-0.190  -0.072-0.119  -0.091-0.138 | 0.583  0.314  0.625  0.690 |
|  | Obliquus externus | Temporal summation  PPT  CPM relative  CPM absolute | -0.506 (1.221)  0.041 (0.064)  0.042 (0.048)  0.047 (0.058) | -2.899-1.886  -0.084-0.166  -0.052-0.137  -0.067-0.162 | 0.678  0.519  0.380  0.415 |
|  | Obliquus internus | Temporal summation  PPT  CPM relative  CPM absolute | 1.574 (1.223)  0.070 (0.064)  0.011 (0.049)  0.056 (0.059) | -0.823-3.972  -0.056-0.195  -0.084-0.106  -0.059-0.171 | 0.198  0.277  0.819  0.337 |
|  |  | Sensory accuracy |  |  |  |
| CoG  Anterior-posterior | Longissimus L3 | Two Point Discrimination  Graphaesthesia  **Vibration** | -0.370 (0.435)  0.470 (0.455)  **4.918 (2.008)** | -1.225-0.485  -0.425-1.360  **0.981-8.854** | 0.398  0.305  **0.014** |
|  | Longissimus L5 | Two Point Discrimination  Graphaesthesia  Vibration | -0.265 (0.430)  0.300 (2.275)  2.602 (1.810) | -5.550-2.875  -0.830-0.945  -0.946-6.150 | 0.536  0.899  0.151 |
|  | Obliquus externus | Two Point Discrimination  Graphaesthesia  Vibration | 0.135 (0.425)  0.805 (0.450)  2.629 (1.714) | -0.700-0.975  -0.080-1.690  -0.729-5.988 | 0.748  0.074  0.125 |
|  | Obliquus internus | Two Point Discrimination  Graphaesthesia  **Vibration** | -0.255 (0.430)  0.075 (0.455)  **3.448 (1.736)** | -1.105-0.590  -0.820-0.965  **0.045-6.851** | 0.554  0.872  **0.047** |
|  |  | Motor control; spiral tracking test | |  |  |
| CoG  Anterior-posterior | Longissimus L3 | Path  Angular distance_Near  Time_Near | -0.003 (0.049)  0.139 (6.041)  0.013 (0.112) | -0.094-0.100  -11.702-11.980  -0.206-0.232 | 0.951  0.982  0.907 |
|  | Longissimus L5 | Path  Angular distance_Near  Time_Near | -0.031 (0.043)  2.355 (5.456)  -0.034 (0.099) | **-**0.115-0.052  -8.338-13.048  -0.227-0.160 | 0.461  0.666  0.733 |
|  | Obliquus externus | Path  Angular distance_Near  Time_Near | 0.068 (0.041)  3.935 (5.444)  -0.078 (0.098) | -0.012-0.148  -6.735-14.604  -0.271-0.115 | 0.097  0.470  0.425 |
|  | Obliquus internus | Path  Angular distance_Near  Time_Near | 0.004 (0.041)  0.622 (5.452)  0.036 (0.099) | -0.085-0.076  -10.064-11.309  -0.157-0.229 | 0.913  0.909  0.716 |

QST, Quantitative Sensory Testing; PPT, pressure pain threshold; CPM, conditioned pain modulation; Path, the total distance travelled in degrees over one trial, and calculated over each quadrant; Angular distance_Near: the mean of the closest 10% to the closest 90% tracking errors; Time_Near: the mean percentage of time spent at an angular distance closer than 0,1° to 0,9° from the red target point. Statistically significant values are highlighted in bold. *Two Point Discrimination and Graphaesthesia are presented in coefficient x 5, as the tests increase in steps of 5.

Appendix 3 Multivariate analyses of the association between CoG medio-lateral coordinate and clinical assessments

| Dependent variable | Muscle | Independent variable | β^­^_association_ (SE) | 95%CI | P value |
| --- | --- | --- | --- | --- | --- |
|  |  | QST pain variables |  |  |  |
| CoG  Medio-lateral | Longissimus L3 | Temporal summation  PPT  CPM relative  CPM absolute | -0.281 (1.077)  0.043 (0.058)  -0.012 (0.042)  -0.006 (0.050) | -2.392-1.831  -0.071-0.156  -0.094-0.069  -0.104-0.092 | 0.794  0.459  0.767  0.905 |
|  | Longissimus L5 | Temporal summation  PPT  CPM relative  CPM absolute | -0.764 (1.068)  0.045 (0.055)  -0.003 (0.042)  -0.019 (0.050) | -2.858-1.329  -0.062-0.152  -0.085-0.078  -0.116-0.079 | 0.474  0.413  0.935  0.704 |
|  | Obliquus externus | Temporal summation  PPT  CPM relative  CPM absolute | 1.050 (1.035)  0.046 (0.054)  0.011 (0.041)  -0.052 (0.049) | -0.978-3.078  -0.061-0.152  -0.070-0.091  -0.149-0.045 | 0.310  0.399  0.798  0.293 |
|  | Obliquus internus | Temporal summation  PPT  CPM relative  CPM absolute | 0.384 (1.037)  0.041 (0.054)  0.002 (0.041)  -0.038 (0.050) | -1.649-2.417  -0.066-0.147  -0.080-0.083  -0.135-0.060 | 0.711  0.456  0.970  0.451 |
|  |  | Sensory accuracy |  |  |  |
| CoG  Medio-lateral | Longissimus L3 | Two Point Discrimination  Graphaesthesia  Vibration | -0.575 (0.370)  0.295 (0.390)  -1.616 (1.759) | -1.295-0.150  -0.470-1.055  -5.064-1.833 | 0.120  0.453  0.358 |
|  | Longissimus L5 | Two Point Discrimination  Graphaesthesia  Vibration | 0.560 (0.365)  -0.185 (0.390)  -1.511 (1.560) | -0.155-1.270  -0.945-0.690  -4.569-1.547 | 0.124  0.634  0.333 |
|  | Obliquus externus | Two Point Discrimination  Graphaesthesia  Vibration | -0.390 (0.360)  0.115 (0.385)  -2.767 (1.462) | -1.095-0.315  -0.645-0.870  -5.633-0.099 | 0.279  0.770  0.058 |
|  | Obliquus internus | Two Point Discrimination  Graphaesthesia  **Vibration** | 0.025 (0.365)  0.215 (0.390)  **-3.470 (1.485)** | -0.690-0.740  -0.550-0.975  **-6.381--0.558** | 0.949  0.584  **0.019** |
|  |  | Motor control; spiral tracking test | |  |  |
| CoG  Medio-lateral | Longissimus L3 | Path  Angular distance_Near  Time_Near | 0.055 (0.044)  2.209 (5.237)  -0.030 (0.098) | -0.031-0.141  -8.055-12.474  -0.222-0.161 | 0.208  0.673  0.755 |
|  | Longissimus L5 | Path  Angular distance_Near  Time_Near | 0.010 (0.037)  -0.464 (4.638)  0.018 (0.084) | -0.062-0.082  -9.554-8.626  -0.147-0.183 | 0.780  0.920  0.830 |
|  | Obliquus externus | Path  Angular distance_Near  Time_Near | -0.025 (0.035)  1.865 (4.626)  -0.033 (0.084) | -0.094-0.044  -7.201-10.931  -0.020-0.131 | 0.473  0.687  0.696 |
|  | Obliquus internus | Path  Angular distance_Near  Time_Near | -0.011 (0.035)  -0.323 (4.634)  -0.007 (0.084) | -0.080-0.058  -9.406-8.760  -0.171-0.158 | 0.747  0.944  0.938 |

QST, Quantitative Sensory Testing; PPT, pressure pain threshold; CPM, conditioned pain modulation; Path, the total distance travelled in degrees over one trial, and calculated over each quadrant; Angular distance_Near: the mean of the closest 10% to the closest 90% tracking errors; Time_Near: the mean percentage of time spent at an angular distance closer than 0,1° to 0,9° from the red target point. Statistically significant values are highlighted in bold. Two Point Discrimination and Graphaesthesia are presented in coefficient x 5, as the tests increase in steps of 5.

Appendix 4 Multivariate analyses of the association between CoG vertical coordinate and clinical assessments

| Dependent variable | Muscle | Independent variable | β^­^_association_ (SE) | 95%CI | P value |
| --- | --- | --- | --- | --- | --- |
|  |  | QST pain variables |  |  |  |
| CoG  Vertical | Longissimus L3 | Temporal summation  PPT  CPM relative  CPM absolute | 0.025 (0.062)  -0.014 (0.034)  -0.022 (0.024)  -0.015 (0.029) | -1.198-1.248  -0.081-0.052  -0.068-0.025  -0.071-0.042 | 0.968  0.676  0.361  0.610 |
|  | Longissimus L5 | Temporal summation  PPT  CPM relative  CPM absolute | -0.484 (0.617)  -0.010 (0.031)  -0.009 (0.024)  0.009 (0.029) | -1.693-0.726  -0.072-0.051  -0.056-0.037  -0.048-0.065 | 0.433  0.740  0.689  0.766 |
|  | Obliquus externus | Temporal summation  PPT  CPM relative  CPM absolute | 0.336 (0.592)  -0.000 (0.031)  -0.012 (0.023)  -0.017 (0.029) | -0.825-1.497  -0.062-0.061  -0.058-0.034  -0.073-0.039 | 0.570  0.995  0.609  0.544 |
|  | Obliquus internus | Temporal summation  PPT  CPM relative  CPM absolute | 0.601 (0.594)  -0.013 (0.031)  -0.033 (0.024)  0.009 (0.029) | -0.564-1.766  -0.075-0.048  -0.079-0.013  -0.048-0.065 | 0.312  0.672  0.163  0.758 |
|  |  | Sensory accuracy |  |  |  |
| CoG  Vertical | Longissimus L3 | Two Point Discrimination  Graphaesthesia  Vibration | 0.050 (0.215)  0.135 (0.225)  -1.834 (1.048) | -0.365-0.470  -0.305-0.575  -3.890-0.218 | 0.810  0.544  0.080 |
|  | Longissimus L5 | **Two Point Discrimination**  Graphaesthesia  Vibration | **0.425 (0.210)**  -0.060 (0.235)  -1.084 (0.090) | **-0.015-0.835**  -0.500-0.375  -2.855-0.688 | **0.042**  0.787  0.230 |
|  | Obliquus externus | Two Point Discrimination  Graphaesthesia  Vibration | -0.105 (0.205)  0.065 (0.220)  -1.003 (0.832) | -0.510-0.300  -0.370-0.500  -2.634-0.629 | 0.613  0.772  0.228 |
|  | Obliquus internus | Two Point Discrimination  Graphaesthesia  **Vibration** | 0.130 (0.210)  0.070 (0.225)  **-2.395 (0.850)** | -0.285-0.540  -0.370-0.505  **-4.060--0.730** | 0.540  0.761  **0.005** |
|  |  | Motor control; spiral tracking test | |  |  |
| CoG  Vertical | Longissimus L3 | Path  Angular distance_Near  Time_Near | 0.023 (0.027)  -0.075 (3.100)  0.006 (0.058) | -0.029-0.075  -6.152-6.001  -0.108-0.120 | 0.391  0.981  0.921 |
|  | Longissimus L5 | Path  Angular distance_Near  Time_Near | 0.011 (0.021)  0.089 (2.660)  -0.003 (0.048) | -0.031-0.053  -5.125-5.302  -0.097-0.092 | 0.600  0.973  0.954 |
|  | Obliquus externus | Path  Angular distance_Near  Time_Near | -0.006 (0.020)  -1.737 (2.651)  0.037 (0.048) | -0.045-0.033  -6.932-3.459  -0.057-0.131 | 0.765  0.512  0.435 |
|  | Obliquus internus | Path  Angular distance_Near  Time_Near | 0.012 (0.020)  -0.936 (2.658)  0.016 (0.048) | -0.028-0.051  -6.145-4.273  -0.079-0.110 | 0.566  0.725  0.745 |

QST, Quantitative Sensory Testing; PPT, pressure pain threshold; CPM, conditioned pain modulation; Path, the total distance travelled in degrees over one trial, and calculated over each quadrant; Angular distance_Near: the mean of the closest 10% to the closest 90% tracking errors; Time_Near: the mean percentage of time spent at an angular distance closer than 0,1° to 0,9° from the red target point. Statistically significant values are in bold. Two Point Discrimination and Graphaesthesia are presented in coefficient x 5, as the tests increase in steps of 5.
